# Digitale Gesundheitstechnologien – das Recht als Hemmschuh oder Wegbereiter?

**DOI:** 10.1007/s00103-024-03835-3

**Published:** 2024-02-08

**Authors:** Merle Freye, Benedikt Buchner

**Affiliations:** 1 Institut für Informations‑, Gesundheits- und Medizinrecht, Universität Bremen, Universitätsallee GW1, 28359 Bremen, Deutschland; 2 Leibniz Science Campus Digital Public Health (LSC DiPH) Bremen, Bremen, Deutschland; 3 Lehrstuhl für Bürgerliches Recht, Haftungsrecht und Recht der Digitalisierung, Universität Augsburg, Augsburg, Deutschland

**Keywords:** Broad Consent, Dynamic Consent, DSGVO, European Health Data Space, Opt-out, Broad consent, Dynamic consent, GDPR, European Health Data Space, Opt-out

## Abstract

Der potenzielle Nutzen digitaler Gesundheitstechnologien hängt im Bereich der populationsbezogenen Gesundheitsforschung maßgeblich davon ab, ob und in welchem Umfang sich diese Technologien auf eine Verarbeitung personenbezogener Gesundheitsdaten stützen lassen. Allerdings herrscht erhebliche Unsicherheit bei der Anwendung und Auslegung der einschlägigen rechtlichen Regelungen zur Verarbeitung von Forschungsdaten mittels digitaler Gesundheitstechnologien. Die Praxis der Forschungsdatenverarbeitung ist immer noch maßgeblich vom Primat der Einwilligung als Legitimationsgrundlage für eine Datenverarbeitung geprägt, obwohl das Informationsmodell des deutschen und europäischen Gesetzgebers mit seinen ambitionierten Anforderungen an die freiwillige und informierte Einwilligung realitätsfern ist. Auch die Konzepte des Broad Consent, Dynamic Consent und Meta Consent, die Alternativen zur klassischen Einwilligungslösung darstellen, können nicht sämtliche Defizite des Einwilligungsmodells beheben.

Um die informationelle Selbstbestimmung der betroffenen Personen zu gewährleisten und gleichzeitig die Interessen der Forschung im Public-Health-Bereich im Blick zu behalten, muss der Forschungsdatenschutz weiterentwickelt werden. Lösungen müssen dabei nicht nur am Einwilligungsverhalten selbst ansetzen, sondern auch eine Legitimation der Datenverarbeitung ganz ohne Einwilligung in den Blick nehmen oder auf eine unwiederbringliche Aufhebung des Personenbezugs der Daten abzielen. Dieser Diskussionsartikel beleuchtet die ambivalente Rolle des Rechts im Hinblick auf digitale Gesundheitstechnologien und zeigt, dass der oftmals als Hindernis verstandene Gesundheitsdatenschutz – bei entsprechender Weiterentwicklung – durchaus den Weg für digitale Gesundheitstechnologien bereiten kann.

## Abstract (en)

The potential benefits of digital health technologies in population-based health research depend mainly on whether and to what extent these technologies can be based on the processing of personal health data. However, there needs to be more certainty in the application and interpretation of the relevant legal regulations on the processing of research data using digital health technologies. Research practice primarily uses consent as a legitimation basis for data processing, although the information model of the German and European legislator, with its ambitious requirements for voluntary and informed consent, is unrealistic and needs to be revised. Even the concepts of broad consent, dynamic consent, and meta consent, which represent alternatives to the classic consent solution, cannot remedy all the deficits of the consent model.

In order to guarantee the informational self-determination of the persons concerned and, at the same time, keep an eye on the interests of research in the public health sector, data protection for research purposes must be further developed. Solutions should not only be tailored to consent behavior but must also consider the legitimization of research data processing without consent or aim to remove the personal reference of the data irretrievably. To date, the law has only fulfilled its task of striking an appropriate balance between the interests of all stakeholders to a limited extent. However, improvement is in sight, especially given current regulatory initiatives and new legal solutions. This discussion article illustrates the ambivalent role of law: on the one hand, health data protection law is often perceived as an obstacle to innovation, but on the other hand, law can pave the way for digital health technologies if further developed.

## Einleitung

Als empirische Wissenschaft ist die populationsbezogene Forschung in besonderem Maße auf eine umfangreiche Nutzung von Daten angewiesen. Egal ob es um die Erforschung der Bedingungen für Gesundheit und Krankheit, die Interaktion zwischen Menschen und ihrer Umwelt oder die Bewertung der Leistung von Gesundheitssystemen geht: All diese Ziele können nur erreicht werden, wenn möglichst viele Daten frei verfügbar sind, leicht verknüpft und langfristig verfolgt werden können. Erst recht gilt dieser Befund, wenn Public Health für die Gesundheitsforschung auf – regelmäßig datengetriebene – digitale Anwendungen setzt. Neben dem wohl prominentesten Beispiel für digitale Anwendungen in der Forschung – der Corona-Datenspende-App – existieren die unterschiedlichsten Studiendesigns in der Forschung *an* und *mit* digitalen Gesundheitstechnologien, die es ForscherInnen nicht nur erlauben, an Forschungsdaten zu gelangen, die „direkt aus dem Leben“ der ProbandInnen stammen, sondern die auch Rekrutierungsbarrieren senken und mitunter finanzielle sowie personelle Ressourcen einsparen können [1].

Die Verarbeitung personenbezogener Gesundheitsdaten dient gleichermaßen gesundheitsbezogenen öffentlichen Interessen wie sie auch erhebliche Vorteile für den Einzelnen mit sich bringt [2]: Auf individueller Ebene geschieht dies etwa in Form einer Verbesserung von Diagnostik und Behandlung, auf übergeordneter Ebene aber auch in der Form, dass eine breite Datenbasis fundiertere gesundheitspolitische Maßnahmen gewährleistet. Dem Recht kommt dabei die Aufgabe zu, die Interessen aller beteiligten AkteurInnen in einen angemessenen Ausgleich zu bringen. Im Rahmen der populationsbezogenen Gesundheitsforschung muss das Recht einerseits für einen möglichst umfassenden Informationszugang einschließlich einer bestmöglichen Aufbereitung der Datensätze im Sinne der FAIR-Prinzipien^1^ sorgen, andererseits aber auch den Persönlichkeits- und Datenschutz aller betroffenen Personen (PatientInnen, ProbandInnen, NutzerInnen etc.) gewährleisten.

Nach wie vor herrscht allerdings in der Forschungspraxis erhebliche Unsicherheit bei der Anwendung und Auslegung der einschlägigen rechtlichen Regelungen zur Verarbeitung von Forschungsdaten mittels digitaler Gesundheitstechnologien [3]. Bei Datenschutz und Forschungsfreiheit handelt es sich um 2 (Grund‑)Rechte, die seit jeher in Konflikt miteinander stehen und sich nur schwer in einen Ausgleich bringen lassen [4, 5] – und noch immer wird Datenschutz in erster Linie als ein Hemmschuh für populationsbezogene Forschung wahrgenommen [6]. In diesem Diskussionsartikel wird aufgezeigt, dass allerdings mehr und mehr Lösungsansätze zu verzeichnen sind, die dem Datenschutzrecht eine forschungsfreundlichere Prägung geben, wie sie ohnehin von Anfang an in der Datenschutz-Grundverordnung (DSGVO) angelegt war. Im Folgenden werden nicht nur die Ansätze der neuen Einwilligungsmodelle, sondern auch einwilligungsfreie Lösungen und Ansätze, die den Anwendungsbereich datenschutzrechtlicher Regelungen neu definieren, beleuchtet.

## Digitale Souveränität dank Einwilligung?

Die Praxis der Forschungsdatenverarbeitung ist immer noch maßgeblich vom Primat der Einwilligung als Legitimationsgrundlage für eine Datenverarbeitung geprägt [7]. Es herrscht die Auffassung vor, dass auch zu Forschungszwecken Daten vor allem dann verarbeitet werden dürfen, wenn die Verarbeitung von einer dahingehenden Einwilligung der betroffenen Person gedeckt ist. Problematisch ist diese Wahrnehmung schon deshalb, weil die Einwilligung – obwohl an sich *die* typische Ausprägung individueller Selbstbestimmung – keineswegs gewährleisten kann, dass eine von der Einwilligung gedeckte Datenverarbeitung auch tatsächlich der autonomen Willensbildung der betroffenen Person entspricht. Dies beginnt bei der (vermeintlichen) Informiertheit der Einwilligung: Die betroffene Person soll informiert in die Verarbeitung ihrer (sensiblen Gesundheits‑)Daten einwilligen, obwohl die entsprechenden Informationen regelmäßig viel zu umfangreich und kompliziert sind und häufig noch nicht einmal gelesen werden. Gerade bei digitalen Anwendungen ist die Idee der informierten Einwilligung regelmäßig auch dann wenig praxisgerecht, wenn schon aufgrund der technisch vorgegebenen Limitierungen (etwa die Displaygröße von Smartphone, Smartwatch etc.) von vornherein ausgeschlossen ist, dass die äußerst komplexen Vorgänge einer digitalen Datenverarbeitung auch nur ansatzweise über entsprechend detaillierte Informationstexte abgebildet werden können. Hinzu kommt, dass die (Forschungs‑)Zwecke einer Datenverarbeitung mit Fortschreiten des Forschungsprojekts oftmals weiterentwickelt oder um neue Zwecke ergänzt werden und Informationen daher zu Beginn des Projekts gar nicht vollständig erteilt werden können.

Das Informationsmodell des deutschen und europäischen Gesetzgebers mit seinen ambitionierten Anforderungen an eine informierte Einwilligung ist somit in vielerlei Hinsicht realitätsfern. Um die informationelle Selbstbestimmung der betroffenen Personen zu gewährleisten und gleichzeitig die Interessen der Forschung im Public-Health-Bereich im Blick zu behalten, muss das Konzept der Einwilligung weiterentwickelt und es müssen auch Wege jenseits des Primats der Einwilligung als Legitimationsgrundlage für eine Datenverarbeitung beschritten werden. Zwar sind mit den Konzepten des Broad Consent, Dynamic Consent und Meta Consent bereits Alternativen zur klassischen Einwilligungslösung vorhanden, jedoch können auch diese nicht sämtliche Defizite des Einwilligungsmodells beheben, insbesondere mit Blick auf das Erfordernis einer informierten Einwilligung (Informed Consent).

### Broad, Meta und Dynamic Consent

Schon seit Längerem werden besondere Formen der Einwilligung propagiert, um den Problemen der Unbestimmtheit und der Informationsüberflutung im Einwilligungsprozess zu begegnen. So soll das Konzept des *Broad Consent* in erster Linie dem Umstand Rechnung tragen, dass zu Beginn eines Forschungsprojekts oftmals noch nicht im Detail absehbar ist, zu welchen Zwecken personenbezogene Daten im weiteren Verlauf des Forschungsprojekts verarbeitet werden, sodass die Einwilligung der betroffenen Person nicht bestimmt genug gefasst werden kann. Um dennoch eine wirksame Einwilligung abgeben zu können, können bei einem Broad Consent in Anlehnung an Erwägungsgrund 33 DSGVO Information und Einwilligung auch weiter („breiter“) ausfallen und sich auf „bestimmte Bereiche wissenschaftlicher Forschung“ beziehen. Für die praxisrelevante Forschung an Universitätskliniken in Deutschland hat etwa die Medizin-Informatik-Initiative (MII) eine Handreichung zur Ausgestaltung des Broad Consent erstellt [8], die auch von der Datenschutzkonferenz (DSK; [9]) als datenschutzkonform akzeptiert worden ist [10]. Allerdings zeigt eben diese Handreichung mit ihrer immer noch 7‑seitigen Musterinformation [11], dass auch der Broad Consent sehr umfangreiche Informationen für die TeilnehmerInnen bedingt und sich daher letztendlich wieder das Problem einer Informationsüberflutung (Information Overload) stellt [12].

Eine Informationsüberflutung geht regelmäßig auch mit dem Modell des *Meta Consent* einher [13], welches dadurch gekennzeichnet ist, dass Probanden nach einer Beratung entscheiden, für welche Art von Forschungsvorhaben sie in welchem Forschungskontext welche Art von Einwilligung geben möchten und wie und wann sie eine Einwilligung in Zukunft abgeben wollen [14]. Es handelt sich somit um eine Einwilligung bezogen auf zukünftige Einwilligungserklärungen, bei der die zur Auswahl stehenden Einwilligungen optional die klassische spezifische Einwilligung, aber auch den Broad Consent umfassen [14]. Der Meta Consent führt vor allem deshalb zu einem Information Overload, da jeweils beschrieben werden muss, für welche Datenverarbeitungen welche Art von Einwilligung zur Verfügung steht.

Die im Modell des Meta Consent auf einen einzigen Zeitpunkt konzentrierte Informationslast soll im Modell des *Dynamic Consent* vermieden werden. Informationen sollen beim Dynamic Consent über einen längeren Zeitraum hinweg verteilt und es so den TeilnehmerInnen ermöglicht werden, sich je nach individueller Präferenz mehr oder weniger zu engagieren und die eigenen Einwilligungen entsprechend in Echtzeit zu ändern [15]. Wie der Dynamic Consent von Meta oder Broad Consent unterschieden wird, ist bisher wenig transparent, letztendlich dürfte der Dynamic Consent vor allem die Tatsache beschreiben, dass eine technische Infrastruktur verwendet wird, die auf die NutzerInnen zugeschnitten ist und über die sie über einen längeren Zeitraum hinweg neue Einwilligungen abgegeben können [7]. In diesem dynamischen Vorgehen liegt jedoch auch ein entscheidendes Argument gegen den Dynamic Consent, denn bei einer mehrmaligen Interaktion und Information der NutzerInnen ist auch die Gefahr eines Ermüdungseffekts höher – insbesondere wenn die jeweiligen Interaktionen und Informationen zeitlich eng beieinander liegen.

Außerdem können sowohl Broad, Dynamic als auch Meta Consent für sich genommen die grundlegenden Problematiken um die Einwilligung nicht vollständig lösen, denn unabhängig davon, wie oft eine Einwilligung eingeholt wird, bleibt offen, worüber und wie transparent über Datenverarbeitungen informiert werden kann. Insofern lösen auch diese Ansätze nicht die zugrunde liegenden Probleme eines jeden Informed-Consent-Modells – die Überfülle und Überkompliziertheit an Informationen –, sondern konzentrieren diese lediglich auf einen Zeitpunkt (Broad und Meta Consent) bzw. verteilen sie auf mehrere Zeitpunkte (Dynamic Consent).

### Informiertheit neu gedacht

Um das Problem der Informiertheit der Einwilligung sachgerecht zu adressieren, bedarf es einer grundsätzlich anderen Herangehensweise. Im Fokus sollte nicht die Frage stehen, auf welche andere Art und Weise Informationen präsentiert werden könnten, sondern vielmehr die Frage, welche Informationen überhaupt präsentiert werden sollten – und zwar nicht mit Blick auf ein Mindest-, sondern vielmehr mit Blick auf ein Höchstmaß an Informationen. Ganz anders präsentieren sich die bisherigen Ansätze: Die von der MII vorgeschlagene Musterinformation im Rahmen des Broad Consent betrifft immer noch 16 Aspekte, über die ProbandInnen informiert werden sollen [11]. Die Zusammenstellung ist erkennbar von dem Bestreben getragen, schulmäßig alle Aspekte abzudecken, die irgendwie von datenschutzrechtlicher Relevanz sein könnten. Im gleichen Stil richten auch die vom Europäischen Datenschutzausschuss (EDSA; [16]) und der DSK [17] angesprochenen allgemeinen Mindestinformationen für eine informierte Einwilligung den Fokus darauf, worüber *mindestens* informiert werden soll, und enthalten gerade keine Begrenzung nach oben.^2^

Angesichts der immer weiter voranschreitenden Komplexität der Datenverarbeitung, gerade bei digitalen Anwendungen, bedarf es aber viel dringender einer Klärung dahingehend, wie ein Informationskatalog aussehen sollte, der nicht unterschritten, vor allem aber auch nicht *überschritten* werden darf, um einer Überforderung und einem Information Overload entgegenzuwirken. Diese Zielrichtung bedingt, dass die Informationspflichten für eine wirksame Einwilligung verdichtet bzw. reduziert werden müssen. Erforderlich ist eine Grenzziehung dahingehend, welche Informationen eine betroffene Person mindestens benötigt – und maximal verarbeiten kann! –, um ihre informationelle Selbstbestimmung tatsächlich souverän und reflektiert wahrnehmen zu können [18]. Entsprechend wird auch in der juristischen Literatur zu Recht eine „verdichtete Informationspräsentation“ [19] oder eine „Verschärfung der Wirksamkeitsvoraussetzungen“ [20] gefordert. Weniger überzeugen können demgegenüber Ansätze, die die umfangreichen Informationspflichten der Art. 13, 14 DSGVO mit der informierten Einwilligung gleichsetzen und somit davon ausgehen, dass eine wirksame Einwilligung vorliegt, wenn alle Information der Art. 13 und 14 DSGVO bereitgestellt wurden [21–23]. So hat auch der EDSA schon anklingen lassen, dass eine gültige Einwilligung „in informierter Weise“ vorliegen kann, selbst wenn nicht alle Elemente der Art. 13 und 14 DSGVO beim Einholen der Einwilligung genannt werden [16, 24, 25].

Welche Informationen für eine informierte Entscheidung elementar sind, sollte künftig vor allem auch unter Einbeziehung der Erkenntnisse anderer Forschungsdisziplinen geklärt werden. Ein solches interdisziplinäres Vorgehen steht in einer Linie mit den Bemühungen der Europäischen Kommission, empirische Entscheidungsforschung in ihre regulatorischen Aktivitäten miteinfließen zu lassen [26, 27]. Insbesondere die Übertragung verhaltenswissenschaftlicher Theorien auf das Einwilligungsverhalten kann Rückschlüsse auf die zu kommunizierenden Informationen vor der Einwilligung liefern und ist gegenüber dem bisherigen Informationsmodell des europäischen Gesetzgebers zumindest im Ansatz empirisch fundiert. Verwiesen sei hier vor allem auf Ansätze, die die Theorie der Schutzmotivation (Protection Motivation Theory – PMT) von Rogers [28] referenzieren und fordern, dass Informationspflichten anhand der PMT entworfen werden müssen [29–33]. Die Forschung hierzu steht jedoch noch ganz am Anfang und es gibt erst wenige Arbeiten, die *konkrete* Informationspflichten in diesem Sinne konzipieren, beispielsweise zur Cookie-Einwilligung [34] oder zur Einwilligung bei Gesundheits-Apps (siehe Dissertationsschrift von Freye M, Publikation vsl. 2024).

## Einwilligungsunabhängige Lösungsansätze

Selbst in Anbetracht der oben dargestellten Ansätze, der Einwilligung als Erlaubnistatbestand zu mehr Legitimationskraft zu verhelfen, bleibt es fraglich, ob die Einwilligung in der Realität je die rechtlichen Idealvoraussetzungen erfüllen kann – auch mit Blick darauf, dass der hier diskutierte Aspekt der Informiertheit nur *einer* der Problempunkte ist, die die Einwilligung als Erlaubnistatbestand kennzeichnen. Gleichermaßen problematisch ist der Aspekt der Freiwilligkeit einer Einwilligung: Was ist etwa von der „Freiwilligkeit“ einer Einwilligung in die Nutzung von Apps zur Nachverfolgung von Kontakten zu Infizierten (Contact-tracing-Apps) zu halten, wenn ohne diese die Teilnahme am sozialen Leben ganz erheblich eingeschränkt wird? Wie „freiwillig“ ist eine Einwilligung bei medizinischen Forschungsprojekten, wenn die betroffene Person als PatientIn um eine Teilnahme ersucht wird? Wenig kompatibel mit den Bedürfnissen der Forschung ist auch die im Datenschutzrecht angelegte freie Widerrufbarkeit der Einwilligung. Ein solcher Widerruf der Einwilligung erfordert nicht nur aufwändige Mechanismen, um den Widerruf im konkreten Fall im Datenbestand umsetzen zu können, sondern läuft darüber hinaus auch dem Forschungsinteresse an einer stabilen und langfristigen Datengrundlage, die insbesondere für epidemiologische Analysen entscheidend ist, zuwider. Letzteres Problem stellt sich ebenso, wenn und soweit man ein mögliches „Verfallsdatum“ der Einwilligung in Betracht zieht [35]. Zu guter Letzt streiten auch die Aspekte der Datenqualität und einer möglichst vollständigen und damit aussagekräftigen Datenbasis (Repräsentativität) gegen den Weg über die Einwilligung [7].

Vor diesem Hintergrund sprechen gute Gründe für Lösungsansätze, die auf eine Legitimation der Forschungsdatenverarbeitung ganz ohne Einwilligung setzen. Teils finden sich solcherlei Modelle schon im geltenden Recht, hierzulande etwa in der Vorschrift des § 27 BDSG, die die entsprechende Öffnungsklausel des Art. 9 Abs. 2 lit. j DSGVO ausformt. Zulässig ist danach eine Verarbeitung besonderer Kategorien personenbezogener Daten u. a. zu wissenschaftlichen Forschungszwecken auch ohne Einwilligung, wenn die Verarbeitung für diese Zwecke erforderlich ist und die Interessen der verantwortlichen Stelle an der Verarbeitung die Interessen der betroffenen Person an der Nichtverarbeitung der Daten erheblich überwiegen.

Auch andere aktuelle Gesetzgebungsinitiativen setzen auf einwilligungsunabhängige Lösungskonzepte, auf deutscher ebenso wie auf europäischer Ebene. So soll mit dem kürzlich erschienenen Referentenentwurf zu einem deutschen Gesundheitsdatennutzungsgesetz (GDNG-E) insbesondere die Freigabe von Daten der elektronischen Patientenakte (ePA) an das Forschungsdatenzentrum ohne Einwilligung auskommen und lediglich eine Widerspruchsoption (Opt-out) enthalten (GDNG‑E, S. 18, 48). Auf europäischer Ebene beschreibt außerdem die in dem Entwurf für eine Verordnung über den europäischen Raum für Gesundheitsdaten (EHDS-VO-E) vorgesehene Datengenehmigung eine einwilligungsfreie Lösung, die von einer Zugangsstelle für die Sekundärnutzung von Gesundheitsdaten ausgestellt wird – und zwar ohne Einwilligung der betroffenen Person, Art. 46 Abs. 1 EHDS-VO‑E.

Die Legitimationskraft all dieser einwilligungsunabhängigen Lösungen hängt ganz wesentlich davon ab, dass der Wesensgehalt des Rechts auf Datenschutz gewahrt bleibt und umfangreiche technische und organisatorische Maßnahmen zur Wahrung der Grundrechte und Interessen der betroffenen Person vorgesehen werden. Hierzu muss insbesondere eine Widerspruchsoption zählen, die der betroffenen Person ein Opt-out jederzeit und ohne Angaben von Gründen so leicht wie möglich macht [36, 37]. Schon die DSK stellte in der Petersberger Erklärung die Widerspruchsmöglichkeit der betroffenen Person als ein wesentliches Element einer einwilligungsunabhängigen Rechtfertigung heraus [38]. Aus diesem Grund ist es ebenso konsequent wie sachgerecht, dass die oben dargestellten einwilligungsfreien Lösungen, die aktuell für die ePA und im GDNG vorgesehen sind, mit einem entsprechenden Widerrufsrecht verknüpft werden.

Zu weit ging daher auch der ursprüngliche Entwurf der Kommission für die EHDS-VO aus dem Jahr 2022, der keine ausdrückliche Widerspruchsoption enthielt, sodass bereits ein totaler Bedeutungsverlust nationaler Opt-out-Regelungen prognostiziert wurde [39, 40] und damit der Einzelne jeglicher Form einer digitalen Souveränität beraubt worden wäre. Für eine Ergänzung der EHDS-VO um eine Opt-out-Lösung, wie sie dann auch in den zweiten Kompromisstext des Rats aufgenommen worden ist, sprechen nicht zuletzt ganz praktische Erwägungen: Erfahrungsgemäß wird ein Widerspruch ohnehin nur ganz selten ausgeübt [41]^3^ und sollte ein Forschungsvorhaben im Einzelfall aufgrund massenhafter Ausübung des Widerrufs tatsächlich zu scheitern drohen, kommt ausnahmsweise auch noch ein Ausschluss des Widerrufsrechts zur Erfüllung einer im öffentlichen Interesse liegenden Aufgabe nach Art. 21 Abs. 6 Hs. 2. DSGVO in Betracht. Angesichts des mit einem Widerrufsrecht einhergehenden Souveränitätsgewinns für die betroffenen Personen und der Praxistauglichkeit entspricht das Widerrufsrecht genau dem erklärten Ziel des EHDS, einen gemeinsamen Raum zu schaffen, in dem einerseits natürliche Personen ihre elektronischen Gesundheitsdaten leicht und selbstbestimmt kontrollieren können, es andererseits aber auch AkteurInnen aus Forschung und Innovation sowie politischen EntscheidungsträgerInnen ermöglicht wird, diese elektronischen Gesundheitsdaten auf vertrauenswürdige und sichere Weise unter Wahrung der Privatsphäre zu nutzen (EHDS-VO-E).

## Aufhebung des Personenbezugs als Ausweg?

Unabhängig von den zuvor dargestellten Initiativen und Lösungsansätzen kann schließlich auch über den Weg der Datenanonymisierung eine leicht(er) zugängliche Datengrundlage für die Forschung eröffnet werden [42], da die DSGVO nur für die Verarbeitung von personenbezogenen Daten gilt, nicht jedoch für anonymisierte Daten. Gerade in der Public-Health-Forschung ist allerdings der Weg über die Anonymisierung oftmals schon deshalb verschlossen, weil es bei einer unwiederbringlichen Aufhebung des Personenbezugs auch nicht mehr möglich wäre, die einzelnen ProbandInnen für wiederholte Kontaktaufnahmen, Rückfragen oder auch für Informationen über eventuell festgestellte Erkrankungen und Diagnosen kontaktieren zu können [10]. Eine wiederholte Kontaktaufnahme ist gerade bei Langzeitstudien unerlässlich, die auf einem Follow-up-Konzept beruhen, wie etwa die NAKO-Gesundheitsstudie, das Sozio-oekonomische Panel (SOEP) oder die britische Kohortenstudie BCS70. Auch bei einer mehrstufigen Delphi-Befragung, die über eine digitale Plattform geschaltet wird, ist eine Pseudonymisierung unabdingbar, um untersuchen zu können, ob die Befragten ihre Meinung während der verschiedenen Befragungsrunden geändert haben. Sollen Daten aus am Körper getragenen Computern (Wearables) mit weiteren Daten verknüpft werden, erfordert diese Datenverknüpfung ebenfalls eine Pseudonymisierung, die sicherstellt, dass die zusammengeführten Daten dieselbe Person betreffen.

Werden Daten zum Zweck der Rückverfolgbarkeit oder Verknüpfung nur pseudonymisiert, nicht aber anonymisiert, sind diese nach herrschender Meinung als personenbezogen einzuordnen und fallen damit in den Anwendungsbereich des Datenschutzrechts. Möglicherweise löst sich die starre Zweiteilung in anonymisiert und pseudonymisiert zukünftig aber auf, wenn anknüpfend an eine Entscheidung des Gerichts der Europäischen Union (EuG) vom April 2023 die Aufhebung eines Personenbezugs von Daten auch für den Fall der Pseudonymisierung zumindest nicht mehr pauschal auszuschließen ist [43]. Das EuG stellte in dieser Entscheidung fest, dass auch eine Pseudonymisierung unter bestimmten Umständen den Personenbezug von Daten entfallen lassen kann, wenn – wie im entschiedenen Fall – pseudonymisierte Daten an eine dritte Stelle weitergegeben werden, die selbst nicht über die Mittel verfügt, um den Personenbezug herzustellen [43]. Diese „anonymisierende Pseudonymisierung“ [44, 45] ist letztendlich eine konsequente Umsetzung der sog. relativen Theorie im Datenschutzrecht, die der Europäische Gerichtshof (EuGH) schon seiner Entscheidung in der Rechtssache Breyer zugrunde gelegt hat [46] und nach der zur Beurteilung eines Personenbezugs eine Risikoprognose erforderlich ist, die auf die Wahrscheinlichkeit von Reidentifizierungsrisiken *der jeweiligen datenverarbeitenden Stelle* abstellt [47].

Setzt sich dieses Verständnis, wie aktuell vom EuG forciert, in der datenschutzrechtlichen Diskussion durch, eröffnen sich vor allem mit dem Modell der Datentreuhand in ganz weitem Umfang Möglichkeiten einer Datenverarbeitung zu Forschungszwecken. Pseudonymisierte Daten wären dann nur noch für diejenige datenverarbeitende Stelle als personenbezogene Daten einzuordnen, die die Zuordnungsregel für ein Pseudonym vergibt und dieses Pseudonym verwaltet – in der Treuhandkonstellation also die Treuhandstelle selbst. Nicht aber wären diese Daten auch für sonstige datenverarbeitende Stellen, die diese pseudonymisierten Daten (für Forschungszwecke) nutzen, als personenbezogene Daten einzuordnen. Dies gilt jedenfalls dann, wenn es sich bei der Treuhandstelle um eine Stelle handelt, die mit besonderen Vertraulichkeitspflichten und -rechten ausgestattet ist und deshalb mit hinreichender Sicherheit ausgeschlossen werden kann, dass diese Stelle den Personenbezug der pseudonymisierten Daten gegenüber anderen Stellen wieder offenlegt. Entscheidend ist, dass es für die forschenden Stellen keinen rechtlich möglichen Weg geben darf, die Zuordnungsregel zu erfahren, und auch kein anderer praktisch gangbarer Weg besteht, um mit einem verhältnismäßigen Aufwand an Zeit, Kosten und Arbeitskräften an die Zuordnungsregel zu gelangen [43, 44, 48].

## Fazit

Der Befund, dass das Datenschutzrecht für die populationsbezogene Gesundheitsforschung an und mit digitalen Gesundheitstechnologien bislang eher Hemmschuh als Wegbereiter ist, hat durchaus seine Berechtigung. Umso wichtiger sind daher neue Impulse aus Wissenschaft, Gesetzgebung und Rechtsprechung für ein forschungsfreundlicheres Datenschutzrecht.

Im Rahmen des bisherigen Einwilligungsmodells zielen neue Lösungsansätze vor allem darauf ab, jenseits von Broad, Dynamic und Meta Consent Informationspflichten neu zu konzipieren, um sicherzustellen, dass die Betroffenen weder mit Informationen überflutet noch von zu komplizierten Informationen überfordert werden.

Aktuelle Gesetzesvorhaben schlagen hingegen einen Weg ein, der von vornherein nicht über die Einwilligung führt, sondern darauf abzielt, einwilligungsunabhängige Legitimationstatbestände auszugestalten. Die zentrale Rolle der Einwilligung soll abgelöst werden durch andere Formen digitaler Souveränität, wie insbesondere die Möglichkeit des Opt-out. Mit dem GDNG‑E und dem EHDS-VO‑E stehen neue Lösungsmodelle vor der Tür, die das Interesse an einer repräsentativen Datenbasis mit dem Schutz informationeller Selbstbestimmung jenseits des Einwilligungsprimats in einen Ausgleich bringen.

Schließlich kann auch der Personenbezug von Daten neu gedacht werden, wenn die bis dato sehr schematisch gehaltene Zweiteilung von pseudonymisierten und anonymisierten Daten aufgebrochen wird und damit vor allem auch Lösungen über eine Datentreuhand zur Umsetzung in der Praxis verholfen werden kann.

## Footnotes

1 FAIR-Prinzipien: Daten sollten auffindbar („findable“), zugänglich („accessible“), interoperabel („interoperable“) und wiederverwendbar („reusable“) sein.

2 Beide verweisen darauf, dass zumindest informiert werden muss über die Art der verarbeiteten Daten, die Verantwortlichen der Datenverarbeitung, die Zwecke der Verarbeitung und das Widerrufsrecht sowie – falls anwendbar – über eine Auslandsdatenverarbeitung und eine automatisierte Entscheidung.

3 Beispielhaft verwiesen sei hier nur auf die Widerspruchsquote von gerade einmal 2 % aller KrebspatientInnen, die laut Bayerischem Landesbeauftragten für den Datenschutz (BayLfD) Prof. Dr. Thomas Petri in Bayern einer Übertragung ihrer Gesundheitsdaten ins Krebsregister widersprechen [41].
